# Changing ecologies, shifting behaviours: Behavioural responses of a rainforest primate, the lion-tailed macaque *Macaca silenus*, to a matrix of anthropogenic habitats in southern India

**DOI:** 10.1371/journal.pone.0238695

**Published:** 2020-09-23

**Authors:** Ashni Kumar Dhawale, M. Ananda Kumar, Anindya Sinha

**Affiliations:** 1 Wildlife Biology and Conservation, WCS-NCBS Programme, National Centre for Biological Sciences, Bangalore, India; 2 School of Natural and Engineering Sciences, National Institute of Advanced Studies, Indian Institute of Science Campus, Bangalore, India; 3 University of Trans-Disciplinary Health Sciences and Technology, Bangalore, India; 4 Nature Conservation Foundation, Mysore, India; 5 Indian Institute of Science Education and Research Kolkata, Mohanpur, India; 6 Cotton University, Guwahati, India; Centre for Cellular and Molecular Biology, INDIA

## Abstract

With the uncontrolled expansion of anthropogenic modifications of the environment, wildlife species are forced to interact with humans, often leading to conflict situations that have detrimental effects for both wildlife and humans. Such interactions are escalating globally, making it crucial for us to devise strategies for both, the management of conflict and the conservation of these often-threatened species. We studied a case of potentially detrimental human-wildlife interactions between an endemic, habitat-specialist primate, the lion-tailed macaque *Macaca silenus* and resident human communities that has developed in recent years in the Western Ghats mountains of southern India. Primates provide useful model systems to understand the extent and nature of behavioural changes exhibited by wildlife in response to anthropogenic habitats with varying degrees of human influence. We documented behaviours, including foraging and intra-species social interactions, to examine the decisions made by the macaques as they exploited four human-modified habitats, which, for the purpose of this study, have been qualitatively characterised to include structural features of the habitat, type of food resources available and the presence of humans. Access to human-origin food, either cooked or packaged, acquired directly from homes or garbage pits, in the human-dominated habitat appeared to significantly reduce active foraging and searching for food, allowing them to engage in other behavioural activities, such as resting. Furthermore, patterns of reciprocated affiliation dissipated in certain human-dominated habitats, with individuals seeming to have adopted novel behavioural strategies, leading to altered social dynamics in the troop, possibly in response to provisioning. This study thus highlights the importance of understanding behavioural changes displayed by animals in response to human interactions; such knowledge could be crucial for the planning and implementation of management and conservation strategies for endangered species such as the lion-tailed macaque and possibly other wildlife in the increasingly anthropogenic landscapes of the tropical world.

## Introduction

Globally, protected areas designated for the conservation of biodiversity account for a very small percentage of landcover, only about 3.48% [1]. Consequently, wildlife and humans, sharing the same landscape, are often forced to interact with one another and this may lead to conflict situations, wherein one or both species may face tremendous losses. It is, therefore, important to understand the ecology of nonhuman species to explain the persistence of these animals in anthropogenic habitats. It then becomes crucial to examine the behavioural changes displayed by individual animals, as these are often amongst the first responses that a species demonstrates to a changing environment [2].

Habitat-modifications have been shown to favour generalist species [3, 4], and lead to behavioural adaptations across various taxa. Omnivores, such as the black bear *Ursus americanus* and spotted hyena *Crocuta crocuta* in urbanized settings, for example, have drastically modified diets and home-ranges, relying heavily on garbage produced by humans [5, 6]. Avian species typically show patterns of homogenisation, leading to high abundance of a few generalist species in response to human-modified habitats [7]. Primates, especially species of the genus *Macaca*, are known to have adapted to a wide variety of habitats across Asia and Africa [8]. The bonnet macaques of peninsular India, for instance, have traditionally been exposed to human presence and appear to have now behaviourally adapted to anthropogenic habitats and human-provided food [9].

Some species, however, still remain, for the large part, isolated from human habitations while others still have only recently begun to come into contact with people. The lion-tailed macaque is a habitat specialist, primarily frugivorous species [10], with very little exposure to humans [11]. In the Valparai region of the Western Ghats mountains of southern India, this species, due to the establishment of commercial plantations and various other encroachments of its rainforest habitats, has recently become exposed to humans and has even begun to frequent their habitations [12]. Given the relatively recent emergence of this phenomenon, however, the ability of such a species to persist in such drastically altered habitats remains largely undocumented.

We thus sought to explore the novel behavioural changes displayed by members of the Valparai subpopulation of lion-tailed macaques as they traversed various anthropogenic habitats. We specifically chose to examine foraging behaviour as it is fundamental to animal survival [13] and it is well established that many taxa show dietary changes in human-altered habitats, often incorporating or even becoming reliant on anthropogenic foods or livestock [3, 14–16]. We also documented social interactions as macaques are group-living animals displaying complex social behaviours, and social interactions are known to significantly affect foraging behaviours [17, 18]. Additionally, we explored the effects of dominance rank, a feature of social dynamics observed particularly in adult female individuals in many primate species [19–21], on foraging behaviours. Finally, we examined pairwise allogrooming patterns among the adult females of the troop, as male lion-tailed macaques rarely indulged in such social interactions [22].

Given logistical constraints, we followed only one of the four troops that occupy human-dominated habitats in our study area. We aimed to uncover the changes in behavioural frequencies that occurred when the troop occupied four structurally distinct habitats with varying degrees of human influence. Previous studies have observed consistent patterns of reduction in foraging frequencies and increased resting in response of different primate species to habitats where human-use foods, often easily assessible in large quantities, are present [23–25]. We thus expected to observe a similar pattern of reduced foraging frequencies displayed by our study individuals in habitats where human-origin foods were present. Likewise, previous studies have observed an increase in affiliation among members within a troop, as a means of reconciliation, when competition over human-use food resources increases in human-dominated habitats [26, 27]. We thus expected to observe a similar pattern of increased intra-troop affiliation amongst the lion-tailed macaques in specific human-dominated habitats where human-origin food was present.

Our study, thus, presents novel insights into the foraging patterns and intra-troop social dynamics of a habitat-specialist primate species in response to habitat-modifications and the presence of humans under drastically changing ecological regimes.

## Materials and methods

### Study area

The study was carried out on the Valparai plateau 10° 19' 39.22"N, 76° 57' 18.98"E, located in the Anamalai hill range of the southern Western Ghats mountains, in the southern Indian state of Tamil Nadu. An expanse of around 220 km^2^, the plateau is a heterogeneous landscape of wet evergreen rainforest fragments interspersed with tea, coffee and *Eucalyptus* plantations [28]. The Anamalai hills are important for the conservation of the endemic lion-tailed macaque, this being one of the eight remaining locations where the species currently occurs [29].

**Fig 1:**
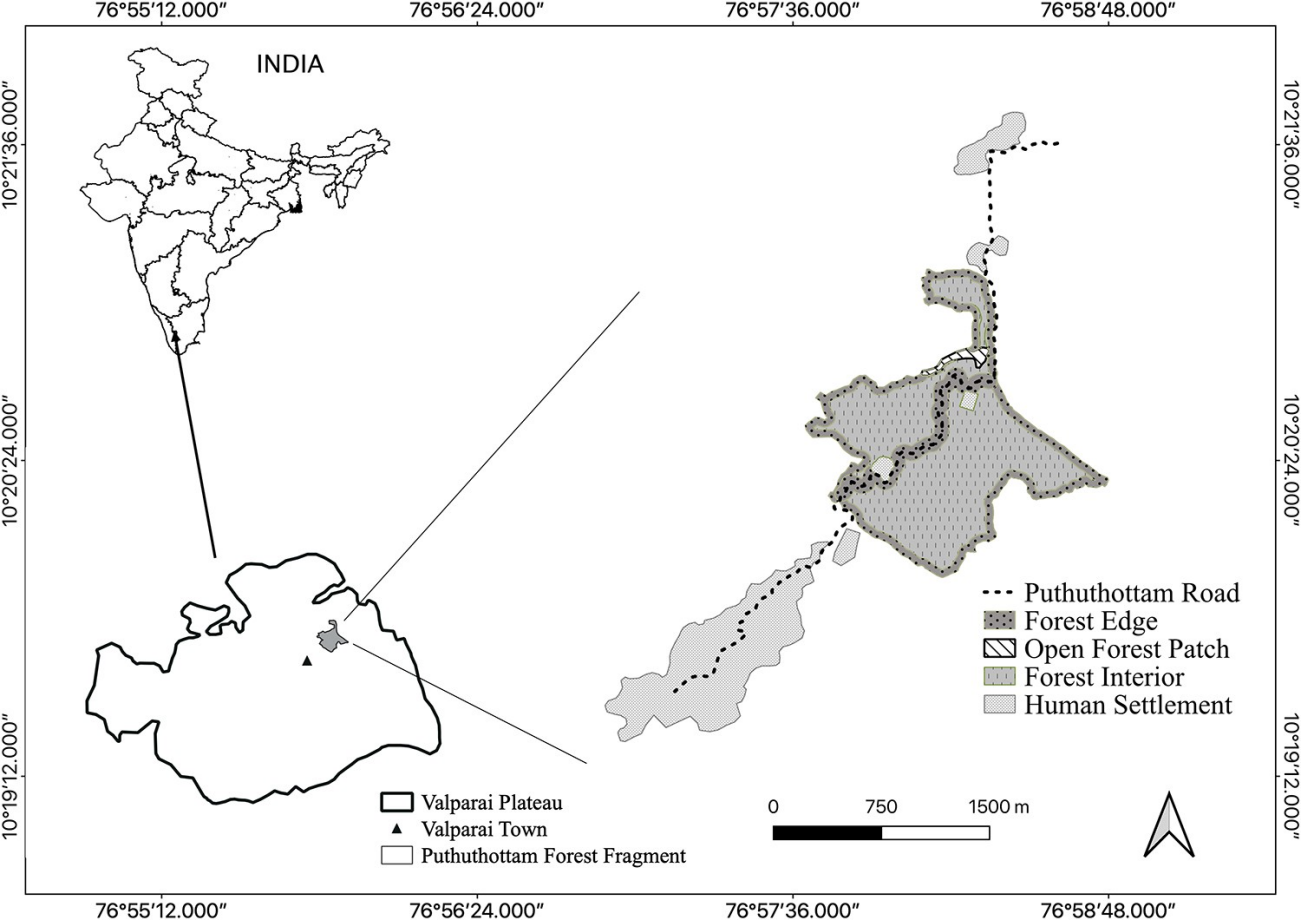
The study habitat types within the Puthuthottam forest fragment, located on the Valparai plateau in the Anamalai Hills, southern India.

**Fig 2:**
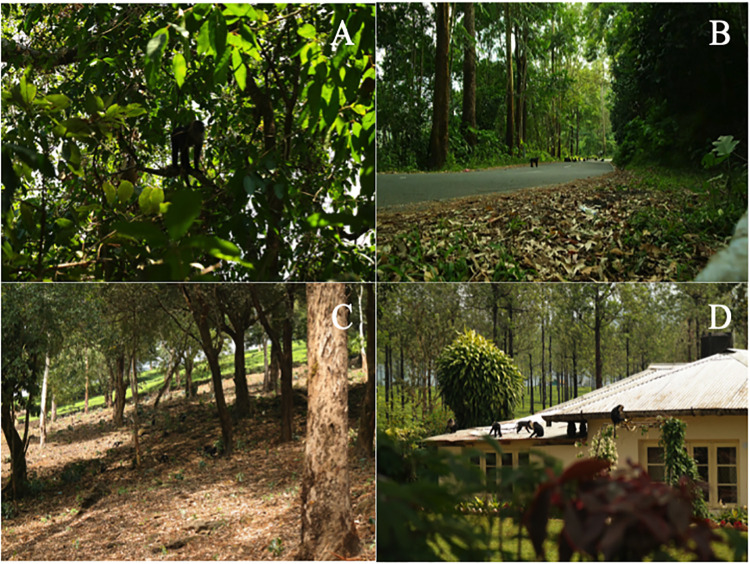
Habitat types present within the Puthuthottam forest fragment: Forest interior (A), Forest edge (B), Open forest patch (C) and human settlement (D).

Since the early 1800s, extensive selective logging has led to the fragmentation and degradation of the native rainforest habitat on the Valparai plateau. One such rainforest fragment, the Puthuthottam forest fragment, with an area of 92 ha and neighbouring the town of Valparai, is of particular importance, as it harbours a subpopulation of macaques with c. 150 individuals (Dhawale, pers. obs). For the purpose of the study, the matrix of habitats present in and around the Puthuthottam forest fragment on the plateau (Figs 1 and 2) were classified as *Forest Edge* (a 50-m-wide belt around the edge of the forest), *Forest Interior* (the area of the forest contained within the aforementioned belt), *Open Forest Patch* (a largely open space within the forest recently planted with coffee saplings) and *Human Settlement*.

### Study species

The lion-tailed macaque *Macaca silenus* a species endemic to the Western Ghats and considered highly specialised to the wet evergreen vegetation type, exists today in 49 subpopulations in eight key locations, one of which is the Anamalai hills [29, 30]. This species is known to be arboreal and primarily frugivorous, making it dependent on the native vegetation of the landscape [10]. In recent years, however, fragmentation and degradation have resulted in the species frequently being exposed to human habitations. Unusually, in some areas, including the Puthuthottam forest, the troops have recently begun to move out of the forest fragment and frequent human habitations [12]. Of the four troops present in Puthuthottam, one particular troop, numbering 92 individuals, has been reported to frequent human habitations at rates as high as 0.43 times a day [12]. While this particular troop has a high human habitation visitation rate, the other three troops do not exhibit such a pattern (Dhawale, pers obs). It is noteworthy that the members of the local human community have reported sighting lion-tailed macaques near their habitations only over the last ten years [31]. For the purpose of our study, we chose one such troop that spent comparable amounts of time in both anthropogenic habitats as well as relatively undisturbed habitats, thus providing a useful model to study the effects of human presence and anthropogenic habitat modification on the study subjects.

### Study troop and individuals

The Puthuthottam macaque subpopulation includes four troops, consisting of c. 92, 37, 15 and 26 individuals respectively. One of these four Puthuthottam troops, the individuals of which could be easily identified and which used all the available human-use habitat types, was selected for this study. The troop consisted of 23 individuals, including nine adult females, one adult male, one subadult male and 12 juveniles (one to six years of age) at the start of the study period, with three infants being born subsequently. The troop was habituated for a period of one month at the start of the study period, during which time all the adult members of the troop were also individually identified using their distinctive features, such as facial markings, injuries or other visible abnormalities, including missing body parts or swellings/bulges.

### Field methods

The study primarily involved the non-invasive following of the selected, habituated troop of lion-tailed macaques, with observations being carried out on all the adult members alone. We conducted our observations for over a total of 480 follow-hours during one of two randomly selected sampling periods (from 08:00 to 14:00 h and 14:00 to 18:00 h) on each day, for 14.5 (± SE of 1) days in a month, for four months, from February to May 2016. We collected data using standard behavioural protocols, including instantaneous group scans for a duration of 5 min each at 15 min intervals, during which time the behavioural states of all individuals were noted (S1 Appendix), and continuous focal animal all-occurrence sampling for foraging and social behaviours, of 10-min duration each conducted on randomly chosen individuals without replacement [32]. Our behavioural sampling effort was 8.80 ± 0.23h per individual, with a total of 96.5 h of focal animal sampling and 217 group scans, consisting totally of 1756 individual scan observations.

All research protocols were reviewed and authorised by the institutional Research Ethics Committee constituted under the Tata Institute for Fundamental Research, Mumbai, India while natural non-invasive observations of the study macaques were conducted with permission from the Tamil Nadu Forest Department (Permit Reference No. WL (A)/ 034559/2015).

### Ecological and behavioural responses of lion-tailed macaques

We defined four different habitats, each of which were qualitatively characterised to include their structural habitats and resource type. The two resource types considered in this study were *Human-origin*, which included all foods such as commercially grown vegetables for cooking, cooked food items, and packaged food items, and *Natural*, which included native (*e*.*g Cullenia exarillata*) and non-native (*e*,*g Spathodea campanulata*) tree species and invertebrates. The four predominant habitat types were identified and characterised during this study, as follows:

#### Forest edge

A 50-m-wide belt around the edge of the Puthuthottam forest fragment, containing native and non-native tree species and bordered on one side by a national highway. We chose to demarcate the boundary at 50m from the edge as we observed that the troop spread at any given time was ≤ 50m. This habitat contained Natural food sources, and occasionally Human-origin foods, either dropped along the roadside or in the form of handouts provided by tourists.

#### Forest interior

An area of forest contained by the Forest Edge, described above, consisting of native and non-native tree species, all of which constituted Natural food sources.

#### Open forest patch

A relatively open space, largely without canopy cover, present within the Puthuthottam forest and recently planted with coffee saplings. It included only Natural food sources.

#### Human settlement

A particular location containing a hospital building, gardens surrounding the hospital and an *Eucalyptus* stand adjacent to the hospital. It was also characterised by the presence of both Natural and Human-origin food resources.

We also recorded human proximity to evaluate changes in behaviour in response to human presence in terms of two distance classes, *0-10m* and *>10m*. Behavioural changes in response to human presence has been presented only for the Human Settlement and the Forest Edge, as humans did not occur in the Forest Interior and very rarely used the Open Forest Patch, not captured in the data.

We described the time-activity budget of the study troop in the four habitat types as the proportion of group scans in which each behavioural state was observed. We compared time-activity budgets across habitat types to investigate whether individuals of the study troop varied their behaviour in response to habitat type.

Foraging behaviour was measured in terms of two behavioural components, *Active Foraging* and *Food Search* (S1 Appendix). Active Foraging behaviours included ingesting of food and rummaging through foliage, dry leaves, bark, or other substratum to acquire food during a feeding bout. Food Search behaviour involved sitting alert and looking around for potential food sources, typically before the start of a feeding bout. During each focal sample, we recorded the duration of Active Foraging/or Food Search behaviours, displayed by the focal individual to the nearest 15 seconds. For the purpose of analysis, we extrapolated the foraging durations from seconds per focal sample of 10-min to minutes per hour and then compared the durations of Active Foraging and Food Search across the four habitat types to uncover the response of lion-tailed macaques to habitat type. Additionally, we compared the foraging durations per hour for two habitat types across the two human proximity distance classes to examine the influence of human presence on the macaques.

Diet composition was measured as the proportion of scan records in which individuals were observed to feed on native and non-native plants (plant materials), and other naturally occurring non-plant matter.

Social behaviour was evaluated in terms of all affiliative and aggressive social interactions, consisting of 34 particular behaviours (S1 Appendix), exhibited by the study individuals and observed during focal animal sampling. These behaviours were grouped into *Affiliation* and *Aggression*, in order to characterise the nature of social interactions displayed by the individuals. We recorded the frequencies of Affiliation and Aggression during focal animal sampling and compared the observed frequencies across habitat types to examine the effects of habitat structure and resource type on the social behaviours displayed by individuals of the study troop. Given the inherently rare occurrence of social interactions in lion-tailed macaques, we were unable to test for the effect of human proximity on these behaviours.

We independently investigated a particularly important affiliative behaviour, *Allogrooming*, which also formed part of Affiliation, defined earlier. Allogrooming was examined in terms of the frequency of initiated allogrooming events per unit time and the duration of allogrooming, as measured by the observed time spent in the behaviour by particular individuals. Another parameter that we calculated was *Reciprocity*, which was defined as the occurrence of any two study individuals directing allogrooming towards one another. We recorded allogrooming initiation frequencies and durations during focal animal sampling, noting down the time to the nearest five seconds. We examined variation in allogrooming by comparing grooming frequencies and time spent grooming across habitats. We created dyadic matrices containing paired (within an individual) measures of grooming initiation and duration, and compared matrices across habitat types to investigate whether these two measures were correlated. This indicated whether the initiation of an allogrooming event led to an allogrooming bout or was initiated without the intention of beginning a grooming bout, purely as a novel affiliation strategy. We created dyadic matrices containing pairwise allogrooming durations for all females in the study group and compared them across habitats to examine whether Reciprocity was specifically observed in human-modified habitats.

Dominance ranks, particularly among adult females, are known to influence patterns of foraging, where dominant individuals often get first access to food sources and other benefits over subordinate females [20]. We established the dominance ranks of all the females, based on approach-retreat behaviours (S1 Appendix) collected during focal animal sampling over the whole study period, thus, each adult female was associated with a single dominance rank. We then examined variations in foraging frequencies of the adult females across dominance ranks within all the habitat types.

### Statistical analyses

All statistical analysis was carried out in R, revised version 3.2.4 [33], with all figures and images also created using R as well.

We established non-normality of data by visually inspecting quantile-quantile (Q-Q) plots while the influence of sampling effort on the response patterns observed was ruled out using the Spearman’s rank-correlation test.

#### Time-activity budgets

We tested for the difference in time-activity budgets across habitat types with the G-test of independence, using the package RVAideMemoire [34].

#### Foraging and social behaviours

We tested for differences in foraging and social behaviours across habitat types using generalised linear mixed-effect models, with individual identity as random effect and the habitat type as fixed effect. We examined differences in foraging behaviour in response to human presence in two human-modified habitats, using a generalised linear mixed-effect model, with an interaction term of habitat type and human proximity as the fixed effect and individual identity as random effect. We also tested for differences in foraging behaviour across dominance ranks for individual females within habitat types using a generalised linear mixed-effect model with dominance rank and habitat type as fixed effects and female identity as random effect. Significance of regression parameters were established using a maximum likelihood ratio test (Laplace Approximation) and models were validated by conducting a visual inspection of residuals using the R package DHARMa [35]. A description of each model with the relevant parameters is presented in Table 1. We added an additional observation-level random effect to each model, in order to account for overdispersion [36]. To investigate individual variation in behaviour within and across habitats, we visually examined the random coefficients from our foraging and social behaviour models. Generalised mixed-effect models were examined using the R package glmmTMB [37].

**Table 1:** Selected models and model parameters for the generalised linear mixed-effect analysis.

*Model*	*Behaviour of Interest*	*Fixed Effect*	*Random Effect*
*1: Habitat effects on Active Foraging*	Active Foraging	Habitat Type	Individual ID
			Observation-level random effect

*2: Habitat and human presence effects on Active Foraging*	Active Foraging	Habitat Type*Human Proximity	Individual ID
			Observation-level random effect

*3: Habitat effects on Food Search*	Food Search	Habitat Type	Individual ID
			Observation-level random effect
*4: Habitat and human presence effects on Food Search*	Food Search	Habitat Type*Human Proximity	Individual ID
			Observation-level random effect
*5: Habitat effects on Affiliation*	Affiliation	Habitat Type	Individual ID
			Observation-level random effect
*6: Habitat effects on Aggression*	Aggression	Habitat Type	Individual ID
			Observation-level random effect
*7: Habitat and dominance rank effects on Active Foraging*	Active Foraging	Dominance Rank*Habitat Type	Individual Female ID
			Observation-level random effect
*8: Habitat and dominance rank effects on Food Search*	Food Search	Dominance Rank*Habitat Type	Individual Female ID
			Observation-level random effect

#### Allogrooming

We used a generalised linear mixed effects model with habitat types as the fixed effect and individual identity as random effect to test for differences in allogrooming frequency and duration across habitat types. We carried out the Mantel test [38] on dyadic matrices of pair-wise measures of allogrooming initiation frequency and duration to test whether allogrooming was being initiated as an affiliative behavioural strategy without the intention of follow-up grooming. We carried out the Mantel test on dyadic matrices of pair-wise allogrooming duration between individuals within each habitat and then compared their correlation across habitats to examine whether Reciprocity was observed in all modified habitats. Mantel tests were carried out using the R package ade4 [39].

#### Dominance rank

We determined the positions of all study individual females within the prevailing linear, transitive, social dominance hierarchy through their behavioural responses during pair-wise approach-retreat interactions [40]. We calculated a dominance score, namely the David’s Score or DS [41], for each female and then categorised them as being *Dominant* (the top three females in the hierarchy), *Subordinate* (the three females with the three lowest ranks in the hierarchy) or *Intermediate* (the three intermediate females in the hierarchy) individuals.

## Results

### Ecological and behavioural responses of lion-tailed macaques to habitat types

The proportion of time spent by the study lion-tailed macaque troop in different habitat types was variable, with 41% of the observed time being spent in the Forest Interior, 21% in the Forest Edge, 9% in the Open Forest Patch and 29% in the Human Settlement.

The time-activity budget of the troop differed significantly between the Forest Interior and the Human Settlement (G-test of independence, G = 84.041, df = 4, p < 0.001)but there were no significant differences between that in the Forest Interior and in the other two habitats.

**Fig 3:**
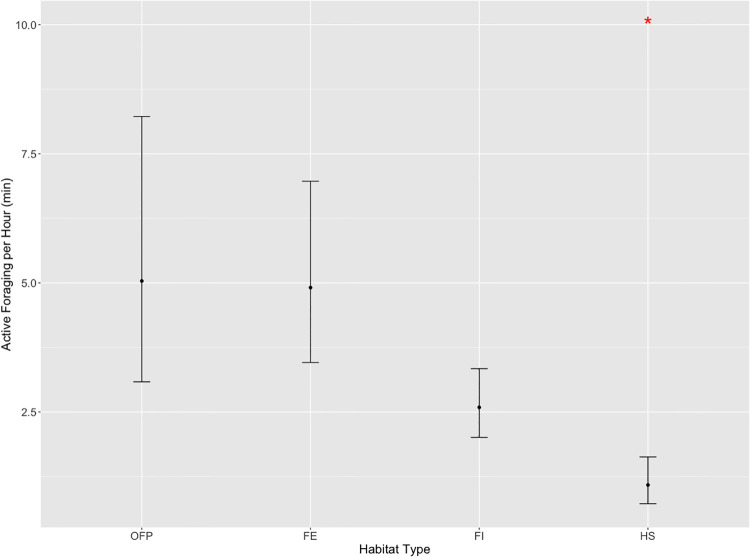
Predicted mean active foraging per hour across habitat types, with 95% confidence intervals. *Statistically significant at p ≤ 0.05. FE = Forest Edge, FI = Forest Interior, OFP = Open Forest Patch, HS = Human Settlement.

**Fig 4:**
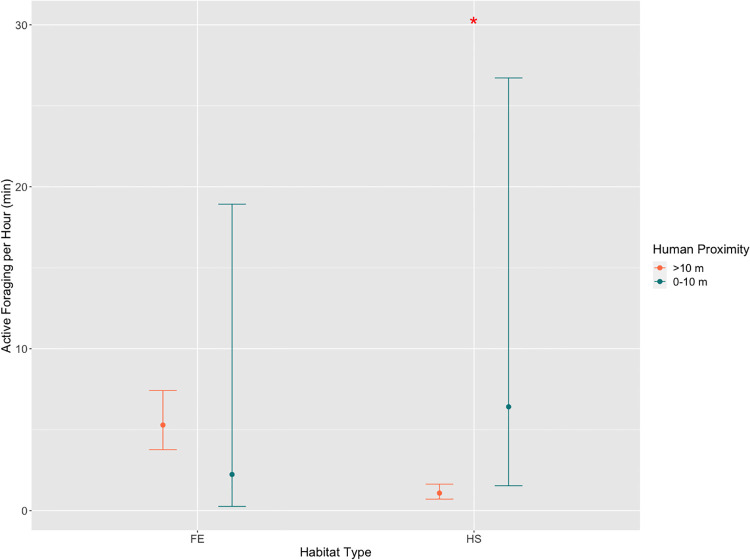
Predicted mean active foraging per hour across human proximity distance classes in two modified habitats types, with 95% confidence intervals. *Statistically significant at p ≤ 0.05. FE = Forest Edge, FI = Forest Interior, OFP = Open Forest Patch, HS = Human Settlement.

Active Foraging displayed by macaques in the Forest Interior (GLMM, *β*_*0*_ = 0.95 ± 0.13, z = 7.30, p < 0.001) increased in the Open Forest Patch (*β*_*1*_ = 0.66 ± 0.28, z = 2.39, p = 0.01) and Forest Edge (*β*_*2*_ = 0.63 ± 0.21, z = 2.95, p = 0.004) but decreased in the Human Settlement (*β*_*3*_ = -0.87 ± 0.23, z = -3.70, p < 0.001; Fig 3). In the Forest Edge, Active Foraging was not significantly higher when humans were >10 m away (GLMM, *β*_*0*_ = 1.66 ± 0.17, z = 9.60, p < 0.001), as compared to when they were within a distance of 10 m (*β*_*1*_ = -0.86 ± 1.10, z = -0.80, p = 0.43). In the Human Settlement, however, Active Foraging was lower when humans were >10 m away (GLMM, *β*_*0*_ = 0.08 ± 0.21, z = 0.30, p = 0.60) but significantly higher when they were within 10 m (*β*_*1*_ = 1.77 ± 0.75, z = 2.4, p = 0.01; Fig 4).

As observed for Active Foraging, Food Search in the Forest Interior (GLMM, *β*_*0*_ = 0.59 ± 0.10, z = 5.48, p < 0.001) was comparable to that in the Open Forest Patch (*β*_*1*_ = 0.34 ± 0.20, z = 1.64, p = 0.10) but significantly lower than that in the Forest Edge (*β*_*2*_ = 0.58 ± 0.15, z = 3.60, p < 0.001) and significantly higher than in the Human Settlement (*β*_*3*_ = -0.86 ± 0.18, z = -4.80, p < 0.001). In the Forest Edge, Food Search did not vary significantly between when humans were >10 m away (GLMM, *β*_*0*_ = 1.21 ± 0.12, z = 9.73, p < 0.001) and when they were within 10 m (*β*_*1*_ = -0.32 ± 0.79, z = -0.4, p = 0.06). In the Human Settlement too, Food Search did not differ significantly when humans were >10 m away (GLMM, *β*_*0*_ = -0.16, ± 0.16, z = -0.97, p = 0.33) or within a distance of 10 m (*β*_1_ = -0.23 ± 1.05, z = -0.07, p = 0.90).

A close investigation of the dietary composition during foraging indicated that the study troop consumed plant parts from a total of 19 species, as well as other non-plant matter (Table 2). The proportion of plant matter in the diet varied significantly between the Forest Interior and the Open Forest Patch (G-test of independence = 60.80, df = 1, p < 0.001) and between the Forest Interior and Forest Edge (G = 16.139, df = 1, p < 0.001) but not between the Forest Interior and the Human Settlement. The troop was thus observed to feed on plant matter significantly more in the Forest Interior than in the Forest Edge and more on invertebrates in the Open Forest Patch than in the Forest Interior.

**Table 2:** Percentage of food species and their parts consumed by the study lion-tailed macaque troop during the study period.

Family	Species	Parts	Percentage
**Lauraceae**	*Litsea floribunda*	Fruits	12.6
**Moraceae**	*Ficus exasperata*	Fruits	11.5
**Moraceae**	*Artocarpus heterophyllus*	Fruits	10.1
**Moraceae**	*Ficus tinctoria*	Fruits	9.5
**Moraceae**	*Ficus racemosa*	Fruits	5.3
**Rubiaceae**	*Coffea liberica*	Fruits	2.9
**Bignoniaceae**	*Spathodea campanulata*	Pods	2.6
**Fabaceae**	*Erythrina indica*	Buds/Pods	2.4
**Moraceae**	*Ficus tsjahela*	Fruits	2.2
**Moraceae**	*Ficus hispida*	Fruits	1.8
**Myrtaceae**	*Syzygium cumini*	Fruits	1.5
**Verbenaceae**	*Lantana camara*	Fruits	1.3
**Myrtaceae**	*Psidium guajava*	Fruits	0.6
**Lauraceae**	*Persea americana*	Fruits	0.3
**Moringaceae**	*Moringa oleifera*	Flower/Buds	0.3
**Rubiaceae**	*Coffea canephora*	Fruits	0.2
**Bombacaceae**	*Cullenia exarillata*	Fruits	0.1
**Passifloraceae**	*Passiflora edulis*	Raw Fruits	0.1
**Others**: Natural food (bark, lichens, fungi, invertebrates) and Provisioned food			34.7

The frequency of Affiliation in the Forest Interior (GLMM, *β*_*0*_ = -8.89 ± 0.95, z = -9.30, p < 0.001) was significantly lower when compared to that in the Open Forest Patch (*β*_*1*_ = 1.18 ± 1.26, z = 0.93, p = 0.30), Forest Edge (*β*_*2*_ = -0.65 ± 1.73, z = -3.80, p = 0.70) and the Human Settlement (*β*_*3*_ = 0.59 ± 1.19, z = 0.50, p = 0.60).

**Fig 5:**
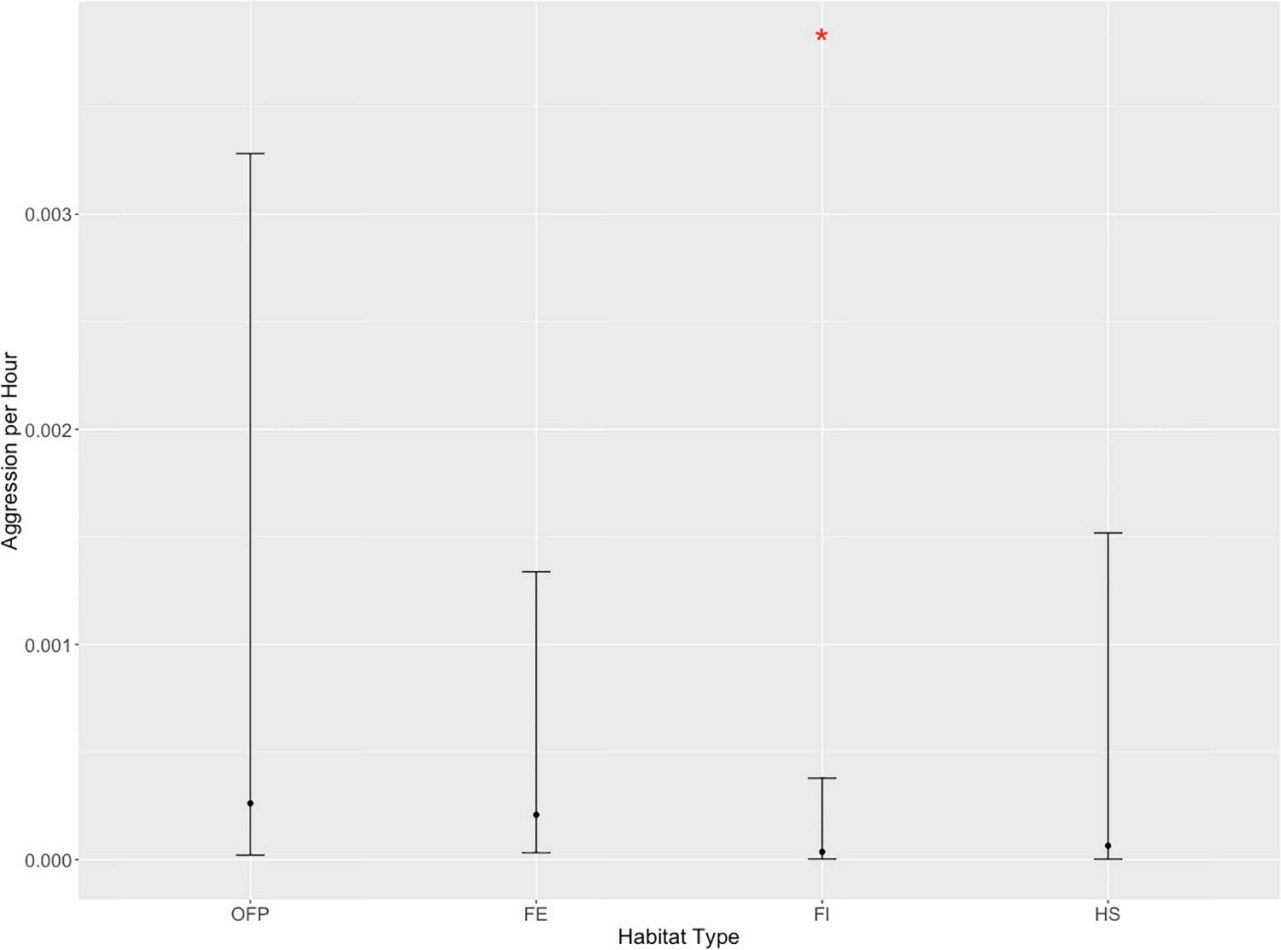
Predicted mean frequency of aggression per hour, across habitats types, with 95% confidence intervals. *Statistically significant at p ≤ 0.05. FI = Forest Interior, FE = Forest Edge, OFP = Open Forest Patch, HS = Human Settlement.

The frequencies of Aggression, as observed in the Forest Interior (GLMM, *β*_*0*_ = -10.23 ± 1,2, z = -8.5, p < 0.001) was significantly lower when compared to the Open Forest Patch (*β*_*1*_ = 1.98 ± 1.47, z = 1.34, p = 0.18), Forest Edge (*β*_*2*_ = 1.75 ± 0.94, z = 1.86, p = 0.06) and Human Settlement (*β*_*3*_ = 0.58 ± 1.76, z = 0.33, p = 0.74; Fig 5).

The observed allogrooming frequencies and durations were comparable across all habitat types (S4 Appendix). The correlation between frequency of initiated allogrooming and allogrooming duration was significantly high in the Forest Interior (Mantel test, correlation coefficient = 0.97, p < 0.001), Forest Edge (0.95, p < 0.001), and Open Forest Patch (0.85, p = 0.0018) but relatively low in the Human Settlement (0.50, p = 0.003).

Reciprocity in allogrooming was observed to be significantly high in the Forest Interior (0.63, p < 0.001), and relatively less so in the Open Forest Patch (0.28, p = 0.04). There was, however, no Reciprocity in allogrooming in the Forest Edge (0.23, p = 0.08) or in the Human Settlement (0.24, p = 0.08).

### Influence of dominance hierarchy on foraging behaviour of female macaques

In the Forest Interior, Active Foraging displayed by Dominant females (GLMM, *β*_*0*_ = 2.30 ± 0.09, z = 24.90, p < 0.001) was comparable to that by Intermediate females (*β*_*1*_ = -0.02 ± 0.13, z = -0.20, p = 0.83), but significantly higher than that shown by Subordinate females (*β*_*2*_ = -0.45 ± 0.13, z = -3.38, p < 0.001). In the Open Forest Patch, in contrast, Dominant females (GLMM, *β*_*0*_ = 1.57 ± 0.16, z = 9.90, p < 0.001) Actively Foraged for significantly less time than did Intermediate females (*β*_*1*_ = 1.19 ± 019, z = 6.22, p < 0.001) or Subordinate females (*β*_*2*_ = 0.98 ± 0.18, z = 5.23, p < 0.001). In the Forest Edge, Dominant females Actively Foraged for less time (GLMM, *β*_*0*_ = 2.22 ± 0.10, z = 21.60, p < 0.001), as compared to Intermediate (*β*_*1*_ = 0.39 ± 0.14, z = 2.74, p = 0.006) or Subordinate females (*β*_*2*_ = 0.41 ± 0.14, z = 2.91, p < 0.001). In the Human Settlement, however, Active Foraging displayed by individuals of Subordinate rank, when compared to that by females of Dominant rank (GLMM, *β*_*0*_ = 1.13 ± 0.14, z = 7.98, p < 0.001) or of Intermediate rank (*β*_*1*_ = -0.06 ± 0.19, z = -0.31, p = 0.75), was significantly higher (*β*_*2*_ = 0.67 ± 0.18, z = 3.66, p < 0.001). It is noteworthy that there was no significant influence of dominance rank on Food Search in any of the four habitats. A closer investigation of individual variation revealed that none of these behaviours appear to be influenced by the Dominance status or the Sex of the individual (S4 Appendix).

### Individual-level behavioural variation in lion-tailed macaques

An examination of the random slopes for Individual identity, a random coefficient in our generalised linear mixed-effects analysis of Active Forage, Food Search, Affiliation and Aggression indicated that neither Dominance Rank nor Sex appeared to explain the variation in the behavioural profiles across the study individuals (S4 Appendix).

## Discussion

With the dramatic, unceasing increase in the expansion of human-dominated landscapes, globally, human-wildlife interactions, which are usually detrimental to all the species involved, are on the rise. In the face of such rapidly changing ecological conditions, behavioural shifts displayed by individual animals are often the first response to environmental change [2] and documenting these responses have become crucial for our understanding of the persistence and management of nonhuman species in these often drastically altered habitats. Habitat-modifications, while occasionally providing new and easily accessible food resources of human origin, have severe detrimental effects, such as increased mortality, even for generalist animal species [42]. Primates, especially macaques, present interesting case studies, as they are extremely ecologically adaptable and capable of co-existing and interacting with humans at rather close quarters. What is often not recognised, however, is that some of these so-called adaptable macaques are now becoming locally extinct [43, 44] and even the common species may have begun to slowly disappear from their well-known, and thus often neglected, habitats [9, 45, 46]. Changes in the demography and behavioural ecology of newly threatened populations of these species, therefore, can be overlooked and fail to garner crucial conservation attention [46, 47].

The lion-tailed macaque is, in many ways, a unique primate, as it is a species that is evolutionarily adapted to the wet evergreen rainforests of the Western Ghats mountains of southern India, a global biodiversity hotspot but, in recent years, some troops of a particular subpopulation of the species has begun to frequent human-dominated areas adjacent to its now-fragmented forest habitats. Consequently, they now interact closely with humans and feed quite often on human-origin resources. This study has, accordingly, documented in some detail the ecological and behavioural patterns exhibited by possibly the most exploratory of these lion-tailed macaque troops as it increasingly explored an anthropogenic landscape over the day and regularly across the months of the year.

A consistent pattern of a drastic decrease in time spent foraging under provisioning regimes, along with an increase in resting behaviour, has been displayed by populations of different species of primates, such as the white-faced capuchin, savanna baboon, rhesus macaque and the barbary macaque [23–25, 48]. A similar pattern was displayed by our study troop when it had access to human-origin resources it reduced foraging significantly and spent an increased amount of time resting. Additionally, the troop would raid the hospital building from anywhere between 20 min to an hour (Dhawale, pers. obs.), following which they would rest in the adjacent *Eucalyptus* stand. Human-origin food, often considered to be of high quality, may occasionally be included by primates in their diet as a strategy to substitute the loss of natural food sources [23]. Consequently, this may result in a troop ultimately becoming dependent on human-origin foods for survival [25], even preferentially gravitating towards areas where human-origin food is regularly available [27, 40]. As it was not possible to collect data during macaque raids on human habitations during this study, a clear estimate of how much of the diet of the study troop consisted of human-origin food could not be established. Evidence from the dietary composition of the troop, however, suggested that such foods was indeed incorporated by individuals as part of their diet. Additionally, in the Open Forest Patch, where much of the under- and mid-storey vegetation has been cleared to plant coffee saplings, the troop consumed a higher amount of invertebrates than they did plant matter, as compared to the other three habitats. Individuals would thus actively forage at high frequencies in this habitat, spending extended periods of time rummaging through dry leaves and bark to acquire insects.

In several studies, the inclusion of provisioned food in the diet of primates have been shown to increase individual fitness, leading to increased reproductive output [49, 50]. Several other studies, however, report detrimental effects of provisioning on the social dynamics, physiological health and survival of nonhuman primates [51–54]. Additionally, when primates are considered pests by humans, especially in the context of home- or garden-raiding, there is increased direct conflict between the two species. Human-nonhuman primate conflict can result in heavy costs to both parties, even, in certain cases, threatening the persistence of the species in a human-dominated landscape [55]. Troops of certain primate species can quickly learn to recognise humans as a potential source of food, even without the actual presence of provisioned foods [25, 56]. In the Forest Edge, during our study, for example, tourists would often stop their vehicles to observe the lion-tailed macaques, subsequently, the macaques learnt to reduced their Active Foraging and Food Search when tourists were within a distance of 10m. Instead, the macaques directed their attention to the vehicles or the tourists, several members moved closer, some even descending onto the road to bipedally survey the vehicles. The adult male particularly approached the vehicles on multiple occasions, this approach almost invariably being followed by tourists attempting to provide food handouts (Dhawale, pers. obs.). Such behavioural associations are known to occasionally lead to individuals running onto roads, putting them at potential risk of accidents [57–59]. In the Human Settlement, humans would often be present within or around the hospital building and the macaques occasionally within 10 m of them. Active Foraging increased under these circumstances, indicating that the macaques were primarily utilising the hospital building, and human presence as indicators, to search for human-origin foods. Long-term studies are, however, needed to provide further information on the degree of qualitative and quantitative dependence on and the patterns of exploitation of human-origin food sources by this population of lion-tailed macaques.

In addition to provisioned food releasing macaque troops from the pressures of intensive foraging, certain studies have reported significant increase in social interactions, with individuals modifying their social strategies under these conditions, bonnet macaques being a notable example [26, 27]. In the case of the study lion-tailed macaques, there was only a marginal increase in frequencies of Affiliation in the Human Settlement. There appeared to be an increase in rates of Aggression in certain habitats, the Open Forest Patch, for example, primarily directed by the adult male towards the subadult male when he attempted to mate with females, a possible manifestation of reproductive competition. The increased aggression in this habitat may have been partly driven by the increased visibility of the troop individuals to one another, a situation not typical in the natural habitat—the rainforest canopy—of the species, allowing the dominant male to detect and react to attempts made by the subadult male to mate with the females. There also appeared to be no increase in the frequency of aggression in the Human Settlement. This was a surprising finding, given that competition over provisioned food is often severe among macaques [26, 27] but it could be argued that these resources were possibly not limiting. This is supported by our observation of a significant increase in the proportion of time spent Resting by the study troop in this particular habitat over the entire study period.

Allogrooming in macaques has been shown to vary as a direct result of human presence, where grooming bouts are shorter and infrequently reciprocated, with individuals also spending less time in grooming under these circumstances [26, 27, 60, 61]. In our study area, the intensity of human activity was relatively low and the troop would usually feed near the buildings before quickly moving on to the adjacent *Eucalyptus* stand, where they would proceed to rest or groom. In lion-tailed macaques, grooming is known to be generally directed up the hierarchy [22]. Our analysis of the patterns of allogrooming in the study troop also revealed that reciprocity in allogrooming, strongly displayed by the study females in forested habitats, broke down significantly in human-dominated habitats. This may have occurred as grooming could have conceivably become a strategy not only to maintain social relationships with other members in the troop but also one to be manipulated in order to reap the benefits of escaping aggression from the most dominant individuals in the troop, as has also been shown earlier in bonnet macaques [26]. Moreover, the observed positive correlation between frequency of initiated allogrooming and the duration of time subsequently spent in it, displayed by the study female lion-tailed macaques in the Forest Interior, Forest Edge and the Open Forest Patch, dissipated in the Human Settlement. This raises the possibility that the macaques initiated grooming more frequently, while reducing time spent in grooming, as a potentially novel social strategy to escape aggression and effect reconciliation under scenarios of increased competition, as prevailed in the anthropogenic habitat, an observation also made earlier in bonnet macaques [26].

Dominance rank is a crucial aspect of the life of social animals, even being shown to affect lifetime reproductive success [21]. Patterns of dominance, especially among the female members of primate troops, become further intensified when food resources occur in a clumped distribution, which is often the case for human-origin foods [27, 62]. In the case of our study troop, subordinate individuals foraged at higher frequencies when compared to dominant individuals in habitats where human-origin foods were present, perhaps as compensation for delayed access to food, privileged first access being reserved for dominant members of the troop.

It is important to note here that our generalised linear mixed-effect analysis of the inter-individual variation in behavioural profiles displayed by the adult individuals in the study troop in certain habitats, including the Forest Edge or Open Forest Patch could not be explained by either sex or dominance rank, the two biological variables that we examined. These variations in behaviour could, however, conceivably be explained by other aspects of the biology of the individual that we did not examine, including, for example, personality or temperament [63]. Such variation, even if not understood completely, could contribute significantly to the ability of individuals to cope with a changing environment. Several questions then beg answers: Are certain individual lion-tailed macaques more likely to initiate novel behavioural strategies and if so, what drives this likelihood? Are these behaviours then learnt by other members of the troop and does such learning lead to the establishment of adaptive behavioural traditions, as has been observed earlier in a population of occasionally provisioned bonnet macaques [64]?

Drivers of primate behavioural ecology, such as human-origin foods and physical human presence, are only some of the factors considered in this study. There are, however, other crucial factors that have not been accounted for in the present investigation. Quantifying the underlying vegetation, for example, may provide a better understanding of the decisions made by the study troop, those that have a clear bearing on the management of potential human-primate conflict and the conservation of this endangered primate species. Additionally, our data represent only the dry season of the year [65] and these behaviours may further vary as availability of resources within the forest change across seasons.

It is widely acknowledged that habitat-modifications affect wildlife of all taxa [66]. Altered habitats tend to favour generalist species, leaving specialist species especially vulnerable to habitat disturbances [67] and this makes it all the more important to investigate the persistence of such species in response to anthropogenic influences. The insights obtained from this study provide a crucial first step in understanding the potential long-term effects that a relatively recent phenomenon, an evolutionarily rainforest-adapted species gradually undergoing a paradigm shift in ecological regimes, through its discovery of an alien, anthropogenic environment may have on the long-term survival of species populations, particularly under conditions of increasing human influence. It may also be strongly suggested that species like the lion-tailed macaque, while being classified as specialist species, seem to have the inherent ability, as do perhaps all macaque species, to adapt to and thrive in human-modified habitats, and this discovery, by itself, may have important implications for the management and conservation of such species populations.

## Supporting information

S1 Appendix An ethogram of the lion-tailed macaque. (DOCX)

S2 Appendix Raw data collected during focal sampling and used for analysis in this study. (XLSX)

S3 Appendix Allogrooming data compiled from the raw data collected during focal animal sampling and used for matrix analysis in this study. (XLSX)

S4 Appendix Allogrooming patterns and random slopes, derived from the generalised linear mixed-effect analysis, depicting behavioural variation across the individuals in the study troop. (DOCX)
