# Supplementary material for: Changing ecologies, shifting behaviours: Behavioural responses of a rainforest primate, the lion-tailed macaque Macaca silenus, to a matrix of anthropogenic habitats in southern India
Source: PLoS One. 2020 Sep 23;15(9):e0238695. doi: 10.1371/journal.pone.0238695 (PMC7511024; doi:10.1371/journal.pone.0238695)
Supplement: S1 Appendix — (DOCX) [file pone.0238695.s001.docx]

**APPENDIX 1: ETHOGRAM**

The ethogram used for this study has been adapted and modified from the unpublished Master’s thesis of Roopali Raghavan (2001). Two earlier studies have presented ethograms for lion-tailed macaques; however, both of these described the behaviour of captively housed groups (Skinner and Lockard 1979; Johnson 1985). The behavioural repertoire of lion-tailed macaques, described here, is based on a troop that ranged in the Puthuthottam fragment of the Valparai Plateau, Anaimalai hills, Tamil Nadu state, from November 2000 to May 2016.

**BEHAVIOURAL STATES** refer to those behaviours that occur over a well-defined period of time. Since these are not instantaneous events, such behaviours are usually measured in terms of the proportion of time that an individual dedicates to them in course of its activity regime. The behavioural states that have been enumerated in the study troop are listed here.

1. *Absent from Troop*: Occasionally, certain individuals may not be with the rest of the troop but could be ranging away from the main activity centres of the group. Care should be taken, however, to ensure that the absence of the individual from the troop is genuine, and that it is not simply at the periphery of the troop and hence, not easily visible to the observer. Adult males in particular were often found to move away from the rest of the troop while foraging in or surveying certain areas of the home range.

2. *Autogroom*: The animal grooms any part on its own body, actively searching for insects, seeds, dirt, ectoparasites or any other object that might be entangled in its hair and focusing its attention on that particular part of the body. This is a common behaviour exhibited by both males and females, but to a relatively large extent by males. In the study troop, adult males displayed a higher frequency of initiated

3. *Drink*: Individuals usually drink by either lying flat, or by simply bending over and placing their mouths directly on the source of water. Occasionally, water may also be scooped out with the hands and drops licked up from the palm. Drinking behaviour is usually observed when the individuals are foraging and chance upon a hollow with water collected therein, or when the troop moves across a stream.

4. *Active Foraging*: This behaviour usually involves the process of handling the food item, active ingestion and the process of chewing while consuming it. Active Foraging can be further classified into *Food Ingestion*, whereby the individual feeds directly from the food source, and *Food Acquisition* which involves the active searching for food items among twigs, branches, green leaves, dry leaves, and hollows on trees as well as on the ground, and the subsequent handling and manipulation of a food item.

5. *Food Search*: This behaviour on the other hand, consists of the individual sitting alertly and looking around for potential food items. Adult females were observed to spend a larger proportion of their time foraging as compared to adult males in the study troop.

6. *Passive Feeding:* This behaviour involves the act of regurgitation of material stored in the cheek pouches and its subsequent ingestion

7. *Move*: Individuals may move continuously along tree branches or on the ground, usually in a specific direction, often not displaying any particular interest to objects along the way. This behaviour was typically observed during troop movements, when the entire group moved from one particular area to another.

8. *Play*: This behaviour involves the acts of mock biting, chasing, lunging, and wrestling between two or more individuals. Although play behaviour was usually observed frequently among infants, juveniles and subadults of both sexes, adult males too occasionally participated in play bouts. Adult females, in contrast, were almost never observed to play, a few exceptions being certain mothers who played with their own infants or with those of other adult females.

9. *Rest*: Individuals usually rest with their feet up on a branch and with the head down on the chest, or by slouching with the head hanging off the branch. During such resting periods, the eyes usually remain open and the individual may be completely awake. Resting has been observed more frequently among adult males than adult females in the study troop.

10. *Sit*: Individuals may sit in a relaxed fashion on any substratum, often without exhibiting a specific interest in any object, individual or event. This has been observed commonly among all individuals of all age and sex classes in the troop.

11. *Sit Alert*: An individual may sit on any substratum and looks attentively at a particular object, individual or event in the surrounding area. This behaviour is exhibited more commonly by adult individuals, with males usually sitting alert more often than do females.

12. *Sleep*: Individuals sleep either in the sitting position or while lying supine – with eyes closed in either situation. Apart from being observed during the late evening or early morning owing to the diurnal nature of the species, sleeping is also met with during afternoons when certain individuals may fall asleep during rests between bouts of foraging.

13. *Stand*: The individual stands on all four limbs on any particular substratum, without necessarily being attentive to any particular object, individual or event in the immediate environment.

14. *Stand Alert*: Individuals may often stand on any substratum and look attentively at a particular object, individual or event in the immediate or distant environment. This behaviour is usually exhibited during inter-troop encounters with members of neighbouring groups or when a predator is sensed.

15. *Survey*: An individual stands attentively, sits alertly or stands erect, bipedal on any substratum, but usually on high branches, and scans the neighbourhood intently. This is a behaviour almost exclusively exhibited by adult and subadult males, although certain dominant females also surveyed the surrounding area on occasions.

**BEHAVIOURAL EVENTS** are instantaneous behaviours or those that occur with a certain frequency, but do not occupy any significant span of time. The behavioural events have been further divided into *individual events* and *social interactions*.

***Individual events*** comprise those behavioural acts that are not directed towards any other individual within the social group. These behaviours may be self-directed, directed towards members of other species, and may be performed in the absence of any other individual or animal.

1. *Alarm Call*: Short gruff bark-like vocalisations are often given when an individual is startled, or in response to alarm calls given by other individuals. Such calls may be directed towards potential predators, as when a raptor flies overhead, or even to human beings. Alarm calls may be given by both males and females, belonging to different age classes.

2. *Body Shake*: An individual, while sitting or standing, may shake its torso vigorously, often to dislodge twigs, fragments of leaves and dust, and also occasionally to shake off water after the rains. This is a common behaviour exhibited by individuals of all age and sex classes.

3. *Cough*: This behaviour, very reminiscent of a human cough, may result from a temporary choking during feeding bouts, during regurgitation, or as a result of a suspected ailment or infection.

4. *Defecate*: Individuals may defecate while sitting or standing on any substratum.

5. *Inspect Penis*: Sexually mature males often inspect their penis, sometimes visually but often with their hands; they may then smell their fingers or lick their palms after such inspection.

6. *Lick Hand*: Individual males and females often lick the palms and fingers of the hand, after the inspection of a bodily wound or of any part of the body.

7. *Masturbate*: Sexually mature males often manipulate their genital organs by rubbing the penis with their hands, often resulting in a visible erection of the penis. Masturbation has been reported by Skinner and Lockard (1979) and Johnson (1985), but neither study has reported ejaculation. Since conditions in field did not facilitate observations of ejaculation, whether masturbation terminates in ejaculation remains uncertain.

8. *Rub Body or Object*: An individual may rub any part of its body or may rub any other object that it is holding against the substratum. An interesting behaviour in this connection is that of rubbing a food item such as a caterpillar against a branch, often after enclosing it within a leaf, in order to remove its hairs.

9. *Scratch Body*: Individuals often scratch different parts of their bodies, sometimes without even being overtly attentive to the act. Scratching is usually performed by individuals of all age and sex classes, although adult males were observed to scratch more frequently than did adult females.

10. *Sneeze*: This behaviour, very reminiscent of the human sneeze, is not restricted to individuals of any particular age or sex class.

11. *Stretch*: The individual stretches its body, while standing. Although not very common, it can be performed by all classes of individuals.

12. *Swipe at Insect*: An individual often vigorously moves its hand in front of its face, presumably to drive away flies or other hovering insects. This is a common behaviour exhibited by all individuals but at higher frequencies during certain seasons or in certain habitats.

13. *Urinate*: An individual urinates while sitting or standing on any substratum.

14. *Wipe Mouth or Nose*: An individual uses its palm to wipe any object or any part of its body; the mouth is often wiped, for example, after an active feeding session.

15. *Yawn*: An individual usually yawns while sitting on any substratum. This behaviour needs to be distinguished from the rather similar-appearing *Bared-teeth Display*, which is usually performed during episodes of aggression. Yawning is usually performed at higher frequencies following periods of rest or sleep.

*Social interactions* include behavioural events that are directed by an individual towards one or more members of the same social group; occasionally such events may also occur between individuals belonging to different, often neighbouring, troops of lion-tailed macaques. Social behavioural events have been further classified into Affiliation, Aggression, Dominance, Play, Sexual Behaviours, Infant-related Behaviours and Neutral Behaviours.

***Affiliation***: This category of behaviours includes those that are employed in an affiliative context during social interactions between individuals and has been used to quantify Affiliation

1. *Affiliative Bared-teeth Face*: This is a communicative gesture in which an individual displays its teeth by pulling apart its lips, with the corners drawn backwards. The mouth is also held partially open. A common behaviour displayed in a variety of non- agonistic situations including, for example, as a greeting display between adult females, it is often accompanied by the *Affiliative Grunt*. This gesture has also been referred to as an Open Bare-teeth Display by Preuschoft (1995)

2. *Affiliative Grunt*: A short grunt-like vocalisation usually made by an individual when encountering another individual in a non-agonistic situation. Exhibited by both males and females alike, it is more common among older individuals and not observed as often among juveniles and infants.

3. *Allogroom*: In this most common of all social interactions, an individual grooms any part of the body of another individual, actively looking for and picking out dirt, ectoparasites and other foreign particles from the hair. Allogrooming is usually performed by individuals of all age and sex classes except infants, who were never observed to allogroom. In the study troop, this behaviour was most frequently directed by adult females towards other females, male-male allogrooming being relatively rare. Occasionally, allogrooming may be accompanied by active lip-smacking.

4. *Contact Call*: A “coo” vocalisation made by an individual, this call is usually made when individuals are moving or foraging in close proximity to each other. Contact calls may or may not, however, receive an immediate response from another individual. Often the contact call may be responded to with a similar-sounding call – this has been referred to as the *Reply Contact Call*. The frequency of contact calls made by adult females was seen to be higher than that given by males in the study troop.

5. *Follow*: One individual may follow a second individual as the latter moves, typically using the same path. This behaviour, used almost invariably with the purpose of approaching the second animal, was displayed by all classes of individuals, but at relatively higher frequencies by infants and juveniles moving with their mothers or siblings.

6. *Friendly Approach*: This refers to an approach made by an individual towards another, followed by the display of any of the affiliative behaviours towards each other.

7. *Hold*: An individual holds another individual by any part of the latter’s body. Although used in an affiliative context, this was of rare occurrence, at least among the members of the study troop.

8. *Hug*: Also referred to by Skinner and Lockard (1979) as an Embrace, the individual stands erect, bipedal or sits and embraces a second individual ventro-ventrally. Like the *Hug*, this behaviour too was of rare occurrence in the study troop.

9. *Long Call*: This is a loud long vocalisation made by an individual when the troop begins to move after spending significant time foraging, feeding or resting in a particular area. Although there may not be an immediate response, the call is sometimes responded to with a similar-sounding call, referred to as the *Reply Long Call*. While adult females gave the long call more frequently than did adult males, the frequency of long calls displayed was observed to be positively correlated to that of *Contact Calls*, described above.

10. *Mouth Touch*: An individual touches the mouth of another individual with its own mouth in an affiliative gesture, although, occasionally the head of the target individual may also be touched. This behaviour is performed by both males and females of all age classes.

11. *Move Away*: The actor moves away from the target individual, terminating an affiliative interaction between the two of them. It must be noted that moving away is never in response to any aggressive act or behaviour displayed by the involved individuals, but a decision made by the actor to simply terminate the affiliative interaction. Although not strictly an affiliative behaviour by itself, this behaviour has been included in this category because it is shown in the context of affiliative interactions alone.

12. *Pat*: The individual gently pats any part of the body of another individual. An affiliative gesture, the pat was rarely performed among the members of the study troop.

13. *Raise Brow*: An affiliative gesture, the actor lifts its eyebrows at another individual, displaying the upper eyelids in the process. The head is also lifted slightly and held up at an angle during the act, while the lifted eyebrows are held in that position for a while. Often repeated in quick succession, this behaviour is usually displayed when an individual approaches another. This appears to be a relatively rare behaviour, observed by Johnson (1985), but not by Skinner and Lockard (1979).

14. *Seek Allogrooming*: An individual often approaches another animal and either lies down, bends, sits or stands in front of the latter, usually looking away from it. This behaviour could either result in the actor being allogroomed by the target individual, or in the latter moving away or otherwise ignoring the request for allogrooming. Observed alike among males and females, higher ranked and larger body-sized individuals in the study troop were more likely to be groomed on seeking allogrooming than were smaller individuals of lower rank.

15. *Seek Support*: During agonistic interactions, one or more of the protagonists may look at an hitherto uninvolved individual and perform an affiliative gesture such as *Affiliative Bared-teeth Face* or *Lip-smack*. The target individual may or may not respond to this behaviour by subsequently participating in the encounter and providing agonistic support to the actor.

16. *Sit Close*: An individual sits at a distance less than half a metre from a second individual. A common affiliative behaviour observed among individuals of all age and sex classes, it usually follows the act of an individual making a *Friendly Approach* towards another individual.

17. *Sit in Contact*: An individual sits with all or some part of its body touching that of a second individual. Although not very common, it has been most frequently observed among certain dyads and particularly during resting periods.

18. *Sleep Together*: The actor sits in contact with or close to the target individual and both fall asleep in this situation. Although common in mother-infant or mother-juvenile dyads, this behaviour is of rare occurrence among adult individuals, particularly adult males.

19. *Touch*: An individual reaches out its hand and touches any part of the body of another individual. This behaviour is often directed toward new or very young infants that arouse the curiosity of other members of the troop.

***Aggression***: This category of behaviours includes those that are employed during agonistic interactions between individuals, and used for the quantification of Aggression

1. *Aggressive Bark*: Sharp bark-like vocalisation that an individual directs towards a target individual during relatively highly aggressive encounters. This call may be given by animals of all age and sex classes.

2. *Aggressive Bite*: The actor may hold tight the body or a body part of the target and bite hard into its flesh. Although occasionally mildly performed during certain within-troop agonistic interactions, the bite may be rather severe during intense aggressive bouts or during inter-troop encounters, often drawing blood in the process.

3. *Bared-teeth Display*: A prolonged deliberate yawn that fully exposes the canines of the individual, this aggressive display gesture is often not directed towards any particular individual. Performed by males much more frequently than by females, this behaviour has also been referred to as a Tension Yawn or Threat Yawn by Skinner and Lockard (1979).

4. *Branch Shake*: Performed more frequently by males than by females, this aggressive gesture consists of an individual holding a branch with its hands and shaking it vigorously. The visual display and the noise produced by this behaviour typically serve to attract attention towards the actor – particularly when performed during within-group and inter-troop agonistic encounters.

5. *Chase*: During aggressive interactions, an individual may rush towards the target individual and follow it closely for some distance. During particularly aggressive encounters, the chase may terminate in an act of contact aggression. The target individual necessarily *Flees* during a chase; this may subsequently be followed by a *Fear Grimace* or *Lip-smack* directed at the chasing individual.

6. *Display Bounce*: An individual stands on a branch and jumps on it causing it to shake vigorously and occasionally break and fall, thus creating a loud commotion during the process. A common display during inter-troop encounters, this behaviour is invariably performed by males and almost always in tense or aggressive situations. A milder version of this act is often by juveniles to initiate play.

7. *Eye Flash*: The individual lifts its eyebrows and pulls back its forehead exposing the upper eyelids in a sudden jerky motion. A behaviour observed only very rarely, it is used in aggressive contexts and is often accompanied by a *Warning Growl* or other aggressive behaviour.

8. *Fear Grimace*: The individual opens its mouth slightly, with the corners of the lips pulled back so as to the expose both rows of teeth. The jaws are not held open and the teeth are parted only very slightly. Displayed by both males and females when receiving aggression, lower ranking individuals perform this gesture much more often than do relatively higher ranked animals. This behaviour has also been referred to as Silent Bared-teeth Display by Skinner and Lockard (1979) and as Grimace by Johnson (1985).

9. *Fear Scream*: A loud high-pitched screech-like vocalisation usually made by an individual in response to received aggression or in fear. This call can be given by individuals of all age and sex classes, but is rarely observed among adult males.

10. *Flee*: An individual runs away quickly from another animal in response to a *Chase* or any other aggression directed towards it. Juveniles and subadults were observed to flee more often than did adults. This behaviour was also noted during inter-troop encounters when individuals of one troop were displaced by those of a more dominant troop.

11. *Lunge*: During certain agonistic interactions, the actor may suddenly rush towards another individual but may stop short before making contact. The torso of the aggressor is often held forward in a directed manner towards the victim of aggression during this behaviour. Displayed most commonly by adult males and females and less often by juveniles, it is almost never exhibited by infants.

12. *Open-mouth Threat*: During this gesture, the mouth of the actor is held wide open displaying the canines for a relatively long duration of time. The head is usually held up such that the chin protrudes forward – towards the victim of aggression. Occasionally, the eyebrows are also raised so as to expose the eyelids. Exhibited by both adult males and females alike, this behaviour is more often directed by higher ranking individuals towards their subordinate counterparts.

13. *Pull Roughly*: An individual forcefully pulls any part of the body of a second individual. This behaviour was invariably used as an aggressive act by members of the study troop, in response to which the recipient was observed to *Fear Scream*, *Fear Grimace* or *Lip-smack*. Johnson (1985) has, however, classified a similar behaviour as one used to ‘reduce tension’ during agonistic interactions; the display of this behaviour apparently involved the pulling of another individual followed by acts of presenting or lip-smacking.

14. *Push Away*: The actor roughly pushes another individual or any part of its body away from itself, often in an effort to avoid unwanted attention. Performed by both males and females, this behaviour is frequently used by adult females to keep away other individuals from their infants. On rare occasions, it was used in the context of active aggression whereby an animal approached another and pushed it away roughly.

15. *Strike with Contact*: A hard hit or slap delivered by the actor on any part of the body of the target individual. Occasionally, a strike did not terminate in contact especially when the target was not physically close enough to the actor (*Strike without Contact*). A rare behaviour, the strike was observed only during highly aggressive interactions.

16. *Warning Growl*: A low growl-like vocalisation directed by an individual towards another during agonistic encounters, the warning growl is used by both males and females alike. Larger, more dominant individuals were, however, observed to direct this behaviour at relatively higher frequencies towards smaller lower ranking animals.

***Dominance***: This category of behaviours includes all interactions related to the establishment and maintenance of dominance-subordination relationships. These have been classified into three groups:

(1) Dominance: Behaviours that are indicative of the actor’s relative dominance over the target individual and, accordingly, invariably directed by dominant individuals towards their subordinate counterparts.

(2) Submission: Behaviours that are displayed by subordinate individuals towards their dominant counterparts and are usually indicative (to the observer) of their subordinate status.

(3) Ambiguous: Behaviours that are often exhibited during dominance-subordination interactions, but which remain ambiguous with respect to the direction in which they are displayed.

**Dominance**

1. *Aggressive Approach*: The actor approaches a target individual and subsequently displays an aggressive act or gesture towards this individual. This behaviour was used as an indicator of dominance-subordination relationships since a dominant individual alone, by definition, exhibited aggressive approach towards subordinate individuals.

2. *Approach Retreated From*: The actor approaches the target individual but the latter moves away from the former without any subsequent interaction occurring. A clear indicator of dominance, this spontaneous moving away from the approaching individual is displayed, by definition, only by a subordinate animal.

**Submission**

1. *Crouch*: The actor physically bends down and often lies low on the substratum or otherwise cowers from a second individual. The head is usually held forward during this act and the fore limbs tucked under the trunk. Johnson (1985) has classified this behaviour as a ‘tension reducing action’.

2. *Retreat*: An individual moves away from an approaching animal in order to maintain a definite distance between them or to move away from it altogether. This behaviour is typically observed during dominance-subordination interactions when the subordinate often spontaneously moves away or during agonistic interactions when the victim of aggression retreats from the aggressor.

Ambiguous

1. *Avoid*: The actor moves or turns its body around in order to avoid facing an approaching individual or any other animal in the vicinity. Although subordinate individuals often actively avoid their dominant counterparts, this behaviour may also be exhibited by relatively high ranking individuals in an effort to deflect unwanted attention; for example, females with infants often turned away from lower ranking females to avoid their infants being handled by the latter.

2. *Avoid Mount*: An individual moves away just as a second individual is about to mount it. Both males and females are known to avoid being mounted by certain individuals, but the relationship of this behaviour to dominance-subordination interactions remains unclear.

3. *Lip-smack*: The actor vigorously opens and closes its mouth in rapid succession, pressing the lips firmly against each other during the closed mouth phase of the act – this invariably leads to a clearly audible smacking sound. Although subordinate animals showed a higher frequency of lip-smacking towards their dominant counterparts in the study troop, higher ranking individuals were also observed to lip- smack, particularly while reassuring lower ranking animals. Lip-smacking can also be used as a gesture of appeasement or reassurance during acts of *Allogroom*, *Present*, *Simple Mount* and *Thrusting Mount*.

4. *Present*: The actor approaches and/or stands in front of the target individual and presents its hindquarters to it. In response to this act, the target individual could mount the actor, inspect its genital area or ignore the act completely. Subordinate individuals were usually observed to present to dominant individuals with lip-smacking (*Present with Lip-smacking*). Males and females in the troop were observed to present to individuals of their own sex as well as to those of the opposite sex. In general, females in the study troop presented relatively much more to the males; on some occasions, however, these were of a sexual nature (see *Sexual Mount* below).

5. *Simple mount*: The actor mounts or partly climbs and clasps the hindquarters of the target individual but without any thrusting movement of the abdomen. In the study troop, males simple-mounted each other more often than females did. Juvenile and female-female mounts were more likely to be simple mounts than *Thrusting Mounts*. Occasionally, simple mounts were observed to be accompanied by lip-smacking (*Simple Mount with Lip-smacking*).

6. *Thrusting mount*: An individual mounts the hindquarters of the target individual and accompanies this act with a thrusting motion of its abdomen; often the genitalia of the actor may be rubbed against that of the target. In the study troop, adult males were more likely to thrusting-mount other males and females than were adult females.

Occasionally, the target individual moved away and avoided being mounted by the actor. Subordinate individuals typically accompanied their thrusting mounts of dominant animals with vigorous lip-smacks (*Thrusting Mount with Lip-smacking*).

***Play***: This category includes all acts of play behaviour indulged in by the different individuals of the troop. Play was most common among subadults, juveniles and infants, although certain adult males were occasionally observed to participate in some play bouts. Adult females, in contrast, almost never played except occasionally with their own infants.

1. *Initiate Play*: An individual may charge another individual, jump on a branch in front of it, or mock chase it, occasionally even approaching and striking out playfully at the animal, with the purpose of initiating play with the second individual.

2. *Play Bite*: The individual gently bites another individual on any part of the body, occasionally holding onto it with its mouth for a short period of time. Most bouts of *Play Wrestle* were accompanied by acts of play bite.

3. *Play Chase*: An individual runs at another animal and chases it around playfully. This could terminate in a wrestling bout or in the target individual turning around and play chasing the actor.

4. *Play Face*: The actor opens its mouth wide and holds it in that position for a short period of time, displaying the teeth during the process. This behaviour is often used to *Initiate Play*. The *Affiliative Bared-teeth Face* and the *Play Face*, described here, have been combined together and referred to as Grin by Johnson (1985). Skinner and Lockard (1979) have not mentioned a gesture equivalent to the *Affiliative Bared-teeth Face* but have described a Relaxed Open-mouth Face or Play Face. Preuschoft (1995), on the other hand, has used the term Open-mouth Bared-teeth Display to refer to this particular behaviour.

5. *Play Jump*: The actor jumps at or very close to the target individual in order to initiate play or in the middle of a play session.

6. *Play Slap*: An individual reaches out and strikes at the target individual, with whom it had been playing, during a play session. This behaviour, although not very common, is often followed by a Play Chase between the same pair of individuals.

7. *Play Wrestle*: Two individuals grapple each other and wrestle, often tumbling off in the process. This is a very common behaviour during play sessions.

***Sexual Behaviours***: This category includes all sexual behaviours that occur between sexually mature males and females in oestrus.

1. *Avoid Sexual Mount*: This refers to the act of an oestrus female in moving away from a male as he prepares to mount her in order to avoid being mounted.

2. *Copulatory Call*: A repeated abrupt “ugh” vocalisation made predominantly by the female while the male is copulating with her or just after he dismounts. Most events of *Sexual Mount* are accompanied by the copulatory call.

3. *Copulatory Grimace*: This gesture is usually made by a copulating male when he lifts his head upwards and parts his lips to display the teeth, although the jaws are not opened too wide. This behaviour has been observed during most acts of *Sexual Mount*.

4. *Inspect*: The male lifts the tail of the female and examines her genitalia. Such inspection may be simply visual (*Visual Inspection*), or it may involve the male smelling the genitalia of the female or smelling his fingers after removing some secretions from the female’s genitalia (*Olfactory Inspection*). Occasionally, the male may also taste the secretions that he has collected from the genital region of the female (*Gustatory Inspection*).

5. *Sexual Approach*: This behaviour refers to the approach made by a male towards an oestrus female, occasionally followed a *Sexual Mount*.

6. *Sexual Mount*: This behaviour involves the mounting of an oestrus female by a male who then thrusts his abdomen repeatedly in order to copulate with her. Since the lion- tailed macaque is known to be a multiple-mount ejaculator, not all sexual mounts necessarily terminate in ejaculation. It is, however, extremely difficult to observe the actual act of ejaculation, following a sexual mount, under field conditions.

***Infant-related Behaviours***: This category includes behaviours that usually characterise mother-infant relationships or those between infants and other adult females. Adult males were virtually never observed to show any interest towards infants.

1. *Carry Infant*: An individual carries an infant as it moves from one place to another. In most cases, it is the mother who carries the infant, although as the infant grows older and less dependent on its mother, it may be carried by other adult females or juveniles for short periods of time.

2. *Groom Infant*: The actor allogrooms any part of the body of an infant, actively looking for and picking out dirt, ectoparasites and other foreign particles from the hair.

3. *Hold Infant*: The individual holds the infant close to its own body, supporting it with both hands.

4. *Nuzzle Infant*: The actor takes its face close to the infant and rubs its face against the infant. Although very rare, this behaviour appears to signify an affiliative relationship between the infant and the actor.

5. *Pull Infant*: An individual pulls the infant close to itself either from another individual or from where the infant is sitting or standing. Adult females were often observed to pull their own infants towards themselves – either just prior to moving or during periods of rest.

6. *Push Infant*: The actor pushes away the infant or any part of the body of the infant away from itself. This behaviour was often observed when an infant interfered with its mother’s feeding, or when an infant attempted to reach out for some object held by the actor.

7. *Touch Infant*: An individual reaches out and touches any part of the body of an infant with its hands. One particular, frequently observed variant of this behaviour is the mouth-touch of an infant.

***Neutral Behaviours***: This category of behaviours includes those that appear to be functionally neutral with regard to agonistic and affiliative relationships, dominance- subordination interactions or sexual relationships.

1. *Ignore*: The actor does not any attention or chooses to look away from any act directed towards it by the target individual. This behaviour has often been observed as a response to the acts of *Present*, *Seek Allogrooming* or *Seek Support*.

2. *Look*: An individual looks directly at the target individual, usually making eye contact in the process. Not seen in the context of aggression or seeking support, the underlying motivation for this behaviour remains unclear.

3. *Lost Call*: A long, loud, wail or moan made by an individual that has become isolated from the rest of the troop and appears to be unable to detect any of the group members. Other individuals from the troop almost invariably respond to the lost call immediately by giving long calls (*Reply Lost Call*) – these calls are given until actor finds its way back to the troop.

4. *Loud Call*: A loud vocalisation comprising a long initial part followed by repeated calls of a shorter duration. This vocalisation is given only by adult males when they move away from the rest of the troop.

5. *Neutral Approach*: The actor approaches another individual and sits within a metre of the latter without any further interaction. This approach is not followed subsequently by the display of any affiliative or aggressive behaviour. Neutral approaches were often made in the context of foraging in an effort to reach a particular food resource.

6. *Observe*: An individual looks at another individual casually and follows its actions or movement for a short period of time. This behaviour could be associated with the target individual failing to realise that it was being observed.

7. *Staccato Call*: Loud repeated short “aah” vocalisation, given only by females. This call has been described as a proceptive call given by oestrus females with the purpose of attracting males (Lindburg 1990). In the study troop, however, this call was made by a dominant female when she did not appear to be in oestrus.

8. *Walk Past*: The actor, while moving, walks past the target individual either brushing against it or moving within ½ metre of it. This behaviour is commonly observed when individuals are foraging together at a clumped food resource.

9. *Visual Exploration*: The actor, while sitting, standing or moving looks around, at an unidentified target, perhaps food or to observe the activities of other troop members.
