# Supplementary material for: Changing ecologies, shifting behaviours: Behavioural responses of a rainforest primate, the lion-tailed macaque Macaca silenus, to a matrix of anthropogenic habitats in southern India
Source: PLoS One. 2020 Sep 23;15(9):e0238695. doi: 10.1371/journal.pone.0238695 (PMC7511024; doi:10.1371/journal.pone.0238695)
Supplement: S4 Appendix — (DOCX) [file pone.0238695.s004.docx]

**APPENDIX 4: ALLOGROOMING AND INDIVIDUAL BEHAVIOURAL VARIATION**

**
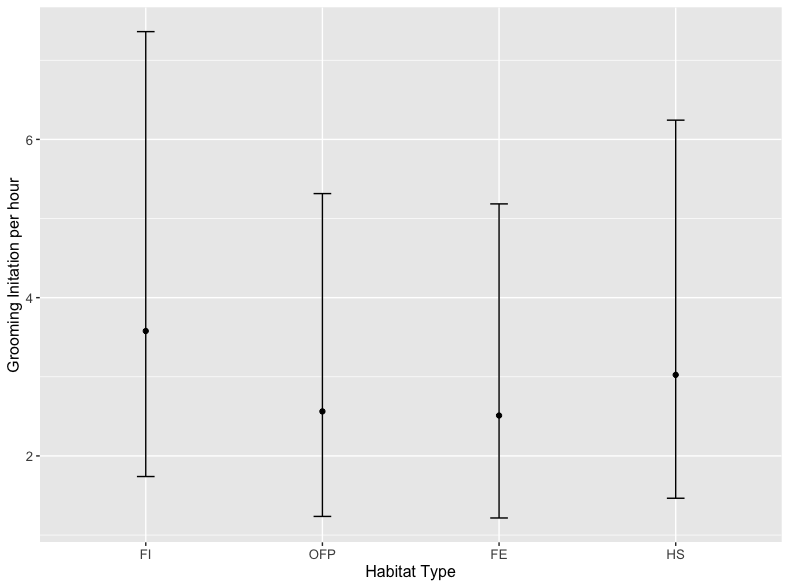
**

**Fig 1.** Predicted mean Allogrooming initiation (frequency per hour) across habitat types, with 95% confidence intervals. *Statistically significant at p ≤ 0.05. FI = Forest Interior , OFP = Open Forest Patch , FE = Forest Edge, HS = Human Settlement


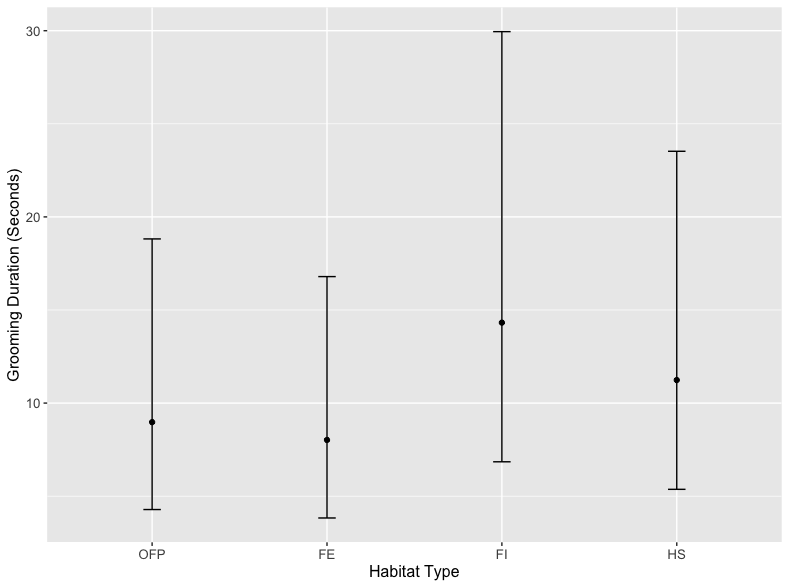


**Fig 2.** Predicted mean Allogrooming duration (seconds) across habitat types, with 95% confidence intervals. *Statistically significant at p ≤ 0.05. OFP = Open Forest Patch, FE = Forest Edge, FI = Forest Interior, HS = Human Settlement

**
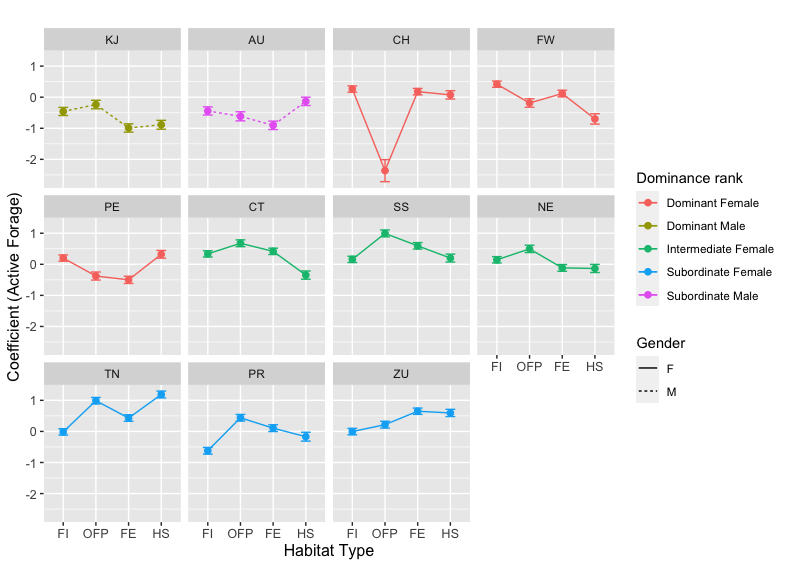
**

**Fig 3.** Individual variation (Mean ± SE) in Active Foraging within and across habitat types. FI = Forest Interior , OFP = Open Forest Patch , FE = Forest Edge, HS = Human Settlement

**
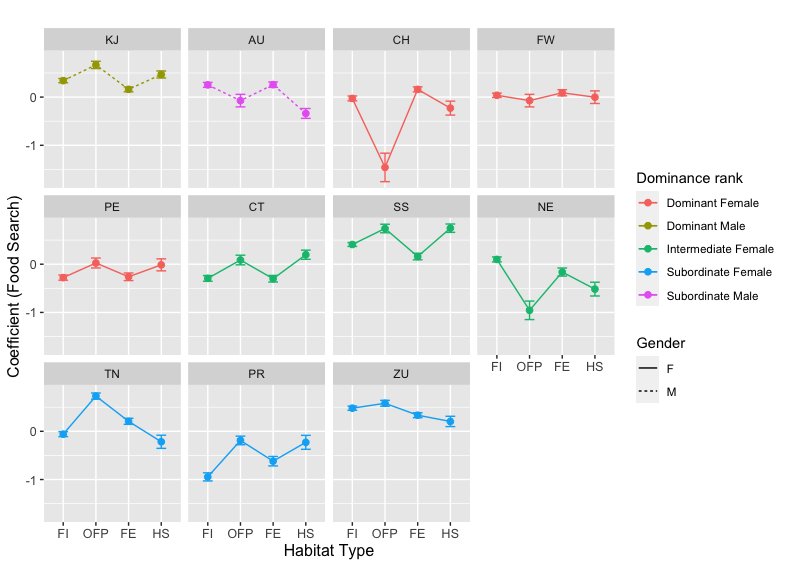
**

**Fig 4.** Individual variation (Mean ± SE) in Food Search within and across habitat types. FI = Forest Interior , OFP = Open Forest Patch , FE = Forest Edge, HS = Human Settlement

**
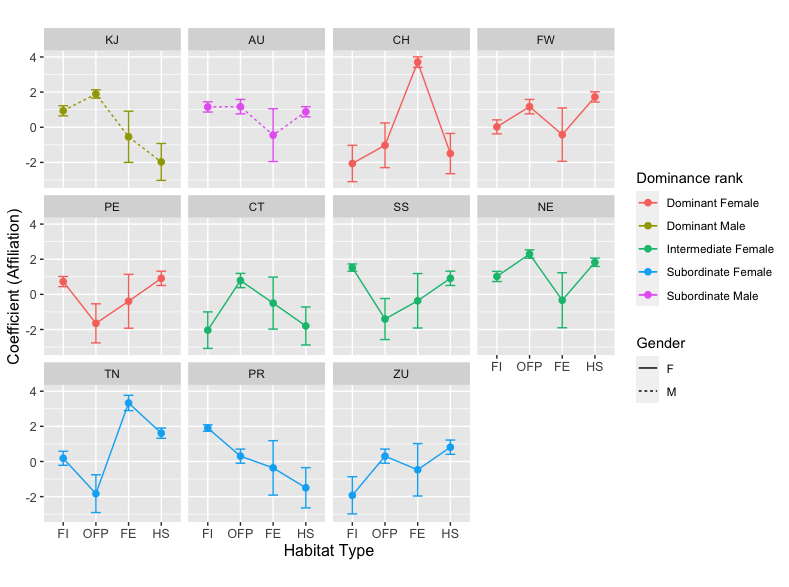
**

**Fig 5.** Individual variation (Mean ± SE) in Affiliation within and across habitat types. FI = Forest Interior , OFP = Open Forest Patch , FE = Forest Edge, HS = Human Settlement

**
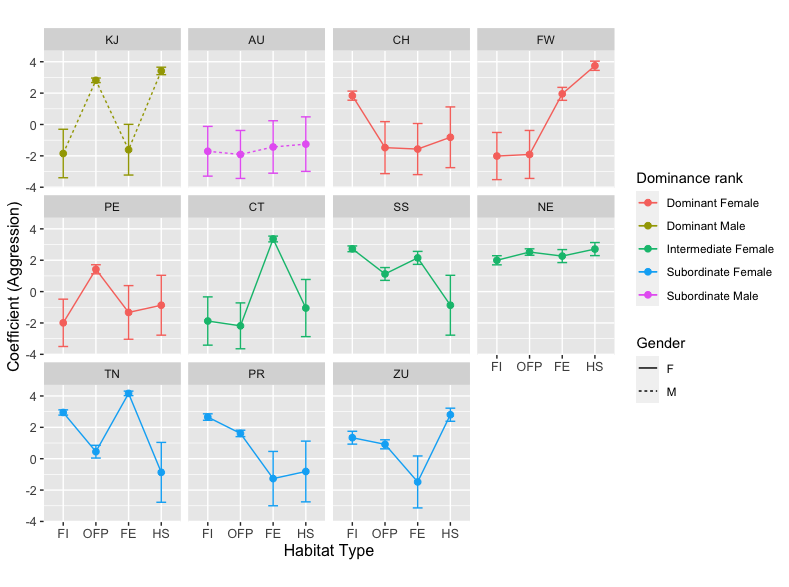
**

**Fig 6.** Individual variation (Mean ± SE) in Aggression within and across habitat types. FI = Forest Interior , OFP = Open Forest Patch , FE = Forest Edge, HS = Human Settlement
